# Off-label use of Baricitinib improves moderate and severe atopic dermatitis in China through inhibiting MAPK and PI3K/Akt/mTOR pathway via targeting JAK-STAT signaling of CD4^+^ cells

**DOI:** 10.3389/fphar.2024.1324892

**Published:** 2024-02-29

**Authors:** Shuang Chen, Caihua Li, Zeng Tu, Tao Cai, Xinying Zhang, Lei Wang, Ruoyuan Tian, Jinglan Huang, Yuxuan Gong, Xiaotong Yang, Zetong Wu, Sirong He, Wenyan He, Dan Wang

**Affiliations:** ^1^ Department of Dermatology, The First Affiliated Hospital of Chongqing Medical University, Chongqing, China; ^2^ Department of Immunology, College of Basic Medicine, Chongqing Medical University, Chongqing, China; ^3^ Chongqing Key Laboratory of Basic and Translational Research of Tumor Immunology, College of Basic Medicine, Chongqing Medical University, Chongqing, China; ^4^ Department of Pathogen Biology, College of Basic Medicine, Chongqing Medical University, Chongqing, China; ^5^ International Medical College, Chongqing Medical University, Chongqing, China; ^6^ Department of Respiratory and Critical Care Medicine, The First Affiliated Hospital of Chongqing Medical University, Chongqing, China

**Keywords:** atopic dermatitis, JAK/STAT, baricitinib, th2, autoimmune diseases

## Abstract

As an inflammatory disease with a disrupted immune system, cytokine disorders in atopic dermatitis (AD) are closely related to the abnormal activation of JAK-STAT signal pathway. The critical relevance of the JAK-STAT signaling pathway to the pathogenesis of AD provides a strong rationale for JAK inhibitor research. Baricitinib, a small-molecule oral JAK inhibitor, has been proven to inhibit JAK-STAT signaling in a variety of diseases, including AD. It is currently available in China for off-label use. However, its efficacy in China and its mechanism are rarely reported. In our study, we found that the immune status of patients with moderate and severe AD was hyperactive. Among the 49 known immunotherapy targets, JAK1 and JAK2 genes on lymphocytes of AD patients were significantly upregulated, which was closely related to the symptom severity in moderate and severe AD patients. Baricitinib can improve immune hyperresponsiveness and clinical symptoms in moderate and severe AD by inhibiting the activation of Th2 cell subsets and the secretion of Th2-type cytokines through MAPK, mTOR and PI3K-Akt signaling pathways, providing an important theoretical basis for clinical off-label use of Baricitinib to treat moderate and severe AD.

## 1 Introduction

Atopic dermatitis (AD) is a relapsing chronic inflammatory skin disease associated with the production of immunoglobulin E (IgE) and secretion of T helper (Th)2 cytokines (Choi and Kim, 2014). This disease affects approximately 15%–20% of children and 1%–3% of adults, and its incidence increases with urbanization (Nutten, 2015; Avena-Woods, 2017). Infiltration of TH2 cells into inflammatory lesions and IgE class conversion in B cells are present in the progress of AD (Raap et al., 2012; Otsuka et al., 2017). The JAK-STAT signaling pathway is a major signaling pathway associated with multiple inflammatory cytokines that regulate immune functions. As an inflammatory disease with a disrupted immune system, cytokine disorders in AD also cause abnormal activation of the JAK-STAT signaling pathway, leading to the development of AD (Rodrigues and Torres, 2020).

There are multiple common traditional treatments for moderate-to-severe AD, but these treatments have different side effects and limitations (Wollenberg et al., 2016). Topical treatment (such as topical corticosteroids and calcium phosphatase inhibitors) and systemic antihistamines are often ineffective in patients with moderate-to-severe AD. Systemic glucocorticoids and traditional broad-spectrum immunosuppressive therapies are often associated with toxic side effects and cannot be used for a long period of time. Moreover, dupleixumab is expensive and not yet widely available in China. Therefore, the treatment of moderate-to-severe AD remains a great challenge for dermatology clinicians.

Baricitinib is a small molecule oral JAK inhibitor that primarily inhibits JAK1 and JAK2, blocking phosphorylation and activation of signal transducers and activator of transcription (STAT), thereby reducing inflammation. The critical relevance of the JAK-STAT signaling pathway to the pathogenesis of AD provides a strong rationale for JAK inhibitor research. In addition, several clinical trials have shown that baricitinib can significantly improve the clinical symptoms of patients with moderate-to-severe AD without significant toxicities (Guttman-Yassky et al., 2019; Reich et al., 2020; Bieber et al., 2021; King et al., 2021; Silverberg et al., 2021; Simpson et al., 2021). Inhibitors of JAK are novel therapies for AD, which are safe and effective with significantly reduced side effects compared to traditional immunosuppressants. Baricitinib was approved in the European Union in 2017 and is currently available in China for off-label use. However, the off-label use of Baricitinib for AD in China and its mechanism are rarely reported.

To investigate the mechanism whereby baricitinib ameliorates the inflammatory response in AD in China, we evaluated the anti-inflammatory and immunomodulatory effects of baricitinib for AD in the clinic and MC903-induced AD mouse models and explored the possible mechanisms by which baricitinib inhibits immune cell-mediated inflammatory responses *in vivo* studies by using AD mouse models and *ex vivo* studies by using digested cells from skin lesions of moderate and severe AD patients. Our study finally revealed that baricitinib inhibited both MAPK and PI3K/Akt/mTOR pathway through JAK-STAT signaling of CD4^+^ T cells, to reduce Th2 cell mediated inflammation, so as to improve moderate and severe AD.

## 2 Methods and materials

### 2.1 Patients and treatment

Main inclusion criteria: a) adults −18–75 years old, and weight of ≥40 kg; b) subject has at least 1-year history of moderate and severe AD. The moderate-to-severe disease was defined as an Investigator’s Global Assessment (IGA) score of three or higher at baseline or an Eczema Area and Severity Index (EASI) score of 12 or higher at baseline according to the American Academy of Dermatology Consensus Criteria and the Hanifin and Rajka criteria (Hanifin and Rajka, 1980; Eichenfield et al., 2014); c) inadequate control/intolerance of topical medications or need for systemic therapy such as oral/injectable medications to control the condition.

Major exclusion criteria: a) subjects with a history of thrombotic events (including deep vein thrombosis, pulmonary embolism, cerebrovascular accidents) or the presence of other known intrinsic states predisposing them to hypercoagulation; subjects with a clear history of infection or neoplasia; b) subjects who have received prior JAK inhibitors (e.g., abrocitinib, baricitinib, upadacitinib, filgotinib, etc.) or systemic therapy with biologic agents; c) subjects who cannot comply with clinical study requirements for visits or cannot sign informed consent; d) Excluding Bullous Pemphigoid, eosinophilic dermatoses and other similar cutaneous diseases; e) subjects with other significant medical conditions that make them unfit to participate in the study.

23 patients with initial moderate and severe AD (mean [SD] age, 51.2 [7.26] years; 78.3% were males), were treated with once-daily 2 mg baricitinib tablets for 4 weeks (Figure 3G; Supplementary Figure S2, S3). The quantitative immunity score (company, patent number), EASI score, vIGA score, and Itch NRS score of each patient were assessed and their peripheral blood was collected at the beginning and end of treatment. Our experiments were approved by The First Affiliated Hospital of Chongqing Medical University Ethical Committee (Ethical approval number: K2023-149).

### 2.2 Animals and treatment

BALB/c mice (*n* = 40, female, weighing 16–18 g, 6–8 weeks old) were housed in a pathogen-free environment with a 12 h light-dark cycle. All the mice were purchased from the Laboratory Animal Center of Chongqing Medical University, and our experiments were approved by Chongqing Medical University Ethical Committee (Chongqing, China). The mice were randomly divided into four groups: normal group (CON), AD model group (AD), Vehicle treatment group (Vehicle), and baricitinib treatment group (Bar). Animals were allowed to acclimate for 1 week before any experimental procedures. The MC903 (a low-calcemic analog calcipotriol of vitamin D3; Sigma, United States) was applied topically to establish an AD mouse model, according to a published protocol (Yeom et al., 2020). Briefly, 2 nmol MC903 (20 μL, dissolved in ethanol) was topically applied to the dorsal side of the right ear, while 20 μL ethanol was applied to the dorsal side of the left ear as solvent control in the AD model group, vehicle treatment group, and baricitinib treatment group once a day for 2 weeks **(** Figure 3A **)**. At the same time, baricitinib (Eli Lilly and Company) was administered to BALB/c mice in baricitinib treatment group daily at 10 mg/kg in 0.5% methylcellulose (Sigma, United States), meanwhile, 0.5% methylcellulose was administered to BALB/c mice in the vehicle treatment group from 8 weeks of age for 2 weeks. Dermatitis scores were evaluated every 2 days.

### 2.3 Scoring of dermatitis, ear thickness

Every 2 days, the ear dermatitis was scored to assess the severity of AD. Dermatitis severity was scored from 0 to 3, which was named as none, mild, moderate, and severe according to the severity of 1) hemorrhage/erythema, 2) itching/crusting, 3) edema, 4) excoriation/erosion Three investigators performed scoring evaluations throughout the study, then calculate the average.

### 2.4 RNA-seq and quantitative immunity score

RNA-seq assays were performed according to the manufacturer’s instructions and were prepared using Illumina TruSeq Stranded mRNA sample preparation kits according to the manufacturer’s protocols. Samples were sequenced on an Illumina NextSeq500 run with single-end 75-bp reads. The raw RNA-Seq data was normalized by reading per kilobase of exon model per million mapped reads (RPKM). The human quantitative immunity score system (Yexin Biotechnology, Chengdu, China) was applied to analyze the immune level of AD patients and the functional levels of immune cell subsets from AD patients according to the manufacturer’s instructions. Sequencing raw data has been uploaded to Sequence ReadArchive in NCBI (https://www.ncbi.nlm.nih.gov/bioproject/PRJNA1041580). Bioproject accession number: PRJNA1041580.

### 2.5 Quantitative real time PCR

Total RNA was extracted from the primary epithelial cells of AD patients and AD mice using a Total RNA Isolation Reagent (Biosharp, Beijing, China). Approximately 1 µg of RNA was reverse transcribed into cDNA using the Reverse Transcription kit (with heat-sensitive double-stranded DNAase) (Biosharp, Beijing, China). 19 μL of SYBR qPCR Mix (Biosharp, Beijing, China) and 1 µL of cDNA were used as templates for real-time fluorescent quantitative PCR. Quantitative SYBR green-based real-time PCR analysis of genes uniquely expressed in lymphocyte subsets was performed using the Lymphocyte Subsets Transcriptional Profiler PCR Array (Yexin Biotechnology, Chengdu, China) on an ABI Prism 7000 System (Applied Biosystems, Foster City, CA, United States) according to the manufacturer’s instructions. The sequences of the qPCR primers used were homo.

FLG (F: 5′-GGA​CAA​CTA​CAG​GCA​GTC​TTG​AA-3′; R: 5′-GTT​TCC​TTC​CTC​TCC​CTC​CTC​TT-3′), homo KRT15 (F: 5′-TAC​AAC​ATG​CTG​CTG​GAC​ATC​AA-3′; R: 5′-TGA​CAC​CAA​TAC​CAG​CCA​TCT​TA-3′), homo TNF-α (F: 5′-CCA​TGT​TGT​AGC​AAA​CCC​TCA​AG-3′; R: 5′-AAG​AGG​ACC​TGG​GAG​TAG​ATG​AG-3′) and homo GAPDH (F: 5′-GGA​GTC​CAC​TGG​CGT​CTT​CA-3′; R: 5′-GTC​ATG​AGT​CCT​TCC​ACG​ATA​CC-3′). qPCR reactions were conducted over 40 cycles of 95°C for 15 s (denaturation), 57.5°C for 30 s (annealing), and 72°C for 30S (extension). Target gene mRNA levels were normalized to glyceraldehyde 3-phosphate dehydrogenase (GAPDH) levels.

### 2.6 Flow cytometry analysis

PBMC from moderate and severe AD patients were separated by density gradient centrifugation using ficoll (Biosharp, Beijing, China) and analyzed by flow cytometry for lymphocyte frequency. Briefly, PBMC were dispersed with 0.25% EDTA-trypsin, washed with PBS, and resuspended in PBS. Subsequently, cells were stained with anti-CD8, anti-CD4, anti-CD44, anti-CD62, and anti-CD69 mAbs incubated for 30 min at room temperature, and then analyzed by flow cytometry using a FACS Calibur system (BD Biosciences, San Diego, CA, United States). Cell sorting and gating strategies are demonstrated in the Supplementary Material (Supplementary Figure S1).

### 2.7 Skin primary cell and PBMC isolation

Skin lesion tissues from AD patients were excised under aseptic conditions, and then the lesion tissues were cut up. The excised skin tissues will be extracted by separation with collagenase and neutral protease (Gibco Laboratories, Grand Island, NY, United States). The free skin lesion tissue cells will be separated by a 100 nm cell filter (Biosharp, Beijing, China). PBMC was isolated through a 75 μm cell filter (Biosharp, Beijing, China). PBMC from AD patients and mice were purified by continuous density gradient centrifugation using Ficoll lymphocyte isolation (Biosharp, Beijing, China).

### 2.8 Cell culture and stimulation

Primary epithelial cells and PBMC from AD patients were cultured in a 6-well transwell chamber (24 mm diameter, 0.4 µm pore size) using a co-culture system in a 5% CO_2_ incubator at 37°C. PBMC (1 × 10^6^) and primary epithelial cells (1 × 10^5^ viable cells/mL) were resuspended in high glucose DMEM (Sigma Corporation, Carlsbad, CA, United States) containing 10% fetal bovine serum (Invitrogen, Carlsbad, CA) and 1% penicillin-streptomycin antibiotic (Invitrogen, Carlsbad, CA) in high sugar DMEM (Sigma, United States). PBMC and primary epithelial cells were then inoculated into the upper and lower chambers of the 6-well transwell chambers, respectively. The co-culture system was treated with baricitinib (55.4 nM; Eli Lilly and Co., Inc.) for 24 h, meanwhile treated with Colivelin TFA (20 μM; MedChemExpress) (Chiba et al., 2005; Zhao et al., 2019), LPS (10 μg/ml; MedChemExpress) (Lai et al., 2017; Dong et al., 2020) and 3BDO (60 µM; TargetMol) (Ge et al., 2014) respectively.

### 2.9 Statistics

All data were expressed as means ± standard deviation (SD). The difference between mean values was determined by Student’s t-test for singular comparisons and by one-way analysis of variance (ANOVA) for multiple comparisons. The area under the curve was determined using the trapezoidal rule. A *p*-value <0.05 was considered significant. All of the treatments were carried out in triplicate.

The materials and methods of Histology and Immunohistochemical Staining, Western Blot, Assessment of cytokines and chemokines are included in **Supplementary 1**.

## 3 Results

### 3.1 Patient disposition, demographics, baseline characteristics, immunity assessment and genetic screening for immunotherapeutic targets in moderate and severe AD patients

Between April 12 and 21 November 2022, 71 patients with initial moderate and severe atopic dermatitis were screened at the Department of Dermatology at the First Hospital of Chongqing Medical University. Patient disposition is summarized in Supplementary Figure S2 (see Supplementary Material). Ultimately 23 patients (mean [SD] age, 51.2 [7.26] years; 18 [78.3%] male) wholeheartedly completed the 4-week treatment period. Mean baseline EASI of the 23 patients was 34.50 (SD 2.41); mean baseline vIGA score was 106.21 (SD 9.64), and mean baseline Itch NRS score was 6.98 (SD 0.47; Table 1).

**TABLE 1:** Demographics, baseline characteristics of the Patients.

	Patients with AD (*n* = 23)
Age (years), mean (SD)	51.2 ± 7.26
Sex	
Male	18 (78.3%)
Female	5 (21.7%)
BMI(kg/m^2^), mean (SD)	25.9 ± 6.8
Quantitative immunity score, mean (SD)	111.23 ± 17.8
EASI score, mean (SD)	34.50 ± 2.41
vIGA score, mean (SD)	106.21 ± 9.64
Itch NRS score, mean (SD)	6.98 ± 0.47

EASI, eczema area and severity index; IGA, Investigator’s Global Assessment, NRS, numerical rating scale.

EASI: The Eczema Area and Severity Index assesses extent of disease at 4 body regions and measures 4 clinical signs: (1) erythema, (2) induration/papulation, (3) excoriation, and (4) lichenification each on a scale of 0–3. The EASI, confers a maximum score of 72.

vIGA: The Validated Investigator Global Assessment used in this study measures the investigator’s global assessment of the patient’s overall severity of their atopic dermatitis based on a static, numeric 5-point scale from 0 (clear) to 4 (severe). The score is based on an overall assessment of the degree of erythema, papulation/induration, oozing/crusting, and lichenification.

Itch NRS: The Itch Numeric Rating Scale is a patient-administered, 11-point horizontal scale anchored at 0 and 10, with 0 representing “no itch” and 10 representing “worst itch imaginable.”

**FIGURE 1:**
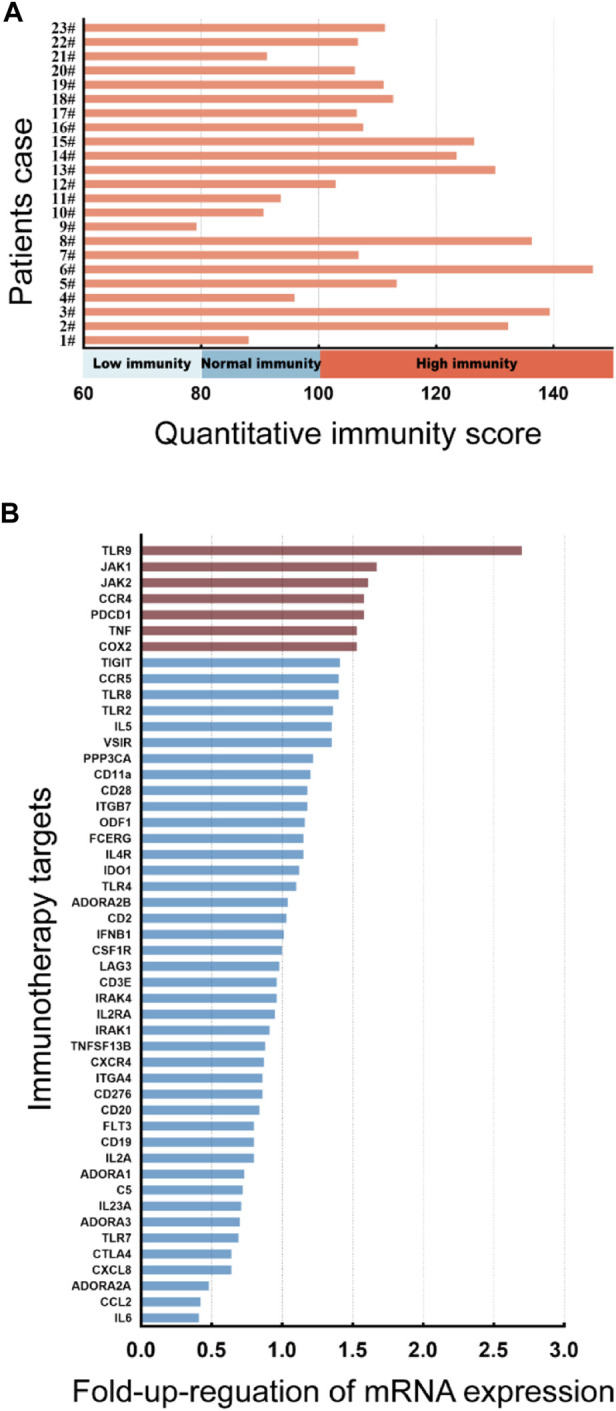
Immunity assessment and genetic screening for immunotherapeutic targets in moderate and severe AD patients. **(A)** The quantitative immunity score of 23 patients with initial moderate and severe AD using the quantitative immunity score system. An immunity score <80 means low immunity, 80<immunity score <100 means normal immunity, immunity score>100 means high immunity. **(B)** Columns represent the fold increase in the expression of 49 immunotherapy target genes in lymphocytes from peripheral blood PBMCs of initial AD patients compared to healthy controls using a real-time PCR array. The figure depicts TLR9, JAK1, JAK2, CCR4, PDCD1, TNF, and COX2 genes exhibiting at least a 1.5-fold upregulation in initial AD patients compared to healthy controls.

To assess the immune status of AD patients, the quantitative immunity score system (Company: Yexin Biotechnology, Chengdu. Patent number: China CN202110987443.4) was applied to evaluate the immune level of 23 patients with initial moderate and severe AD who had completed the treatment period. Compared to the immune level of healthy controls, the results showed that 73.9% of these AD patients (*n* = 17) were at the immune hyperactive level (Figure 1A), indicating that AD is an autoimmune disease with hyper immunity. We further examined the expression levels of 49 known immunotherapeutic targets in lymphocytes from these AD patients. We found that the mRNA expression levels of TLR9, JAK1, JAK2, CCR4, PDCD1, TNF, and COX2 target genes were more than 1.5 times higher than those of healthy controls (Figure 1B).

### 3.2 High-JAK1/2 expression is associated with symptom severity in moderate and severe AD patients

**FIGURE 2:**
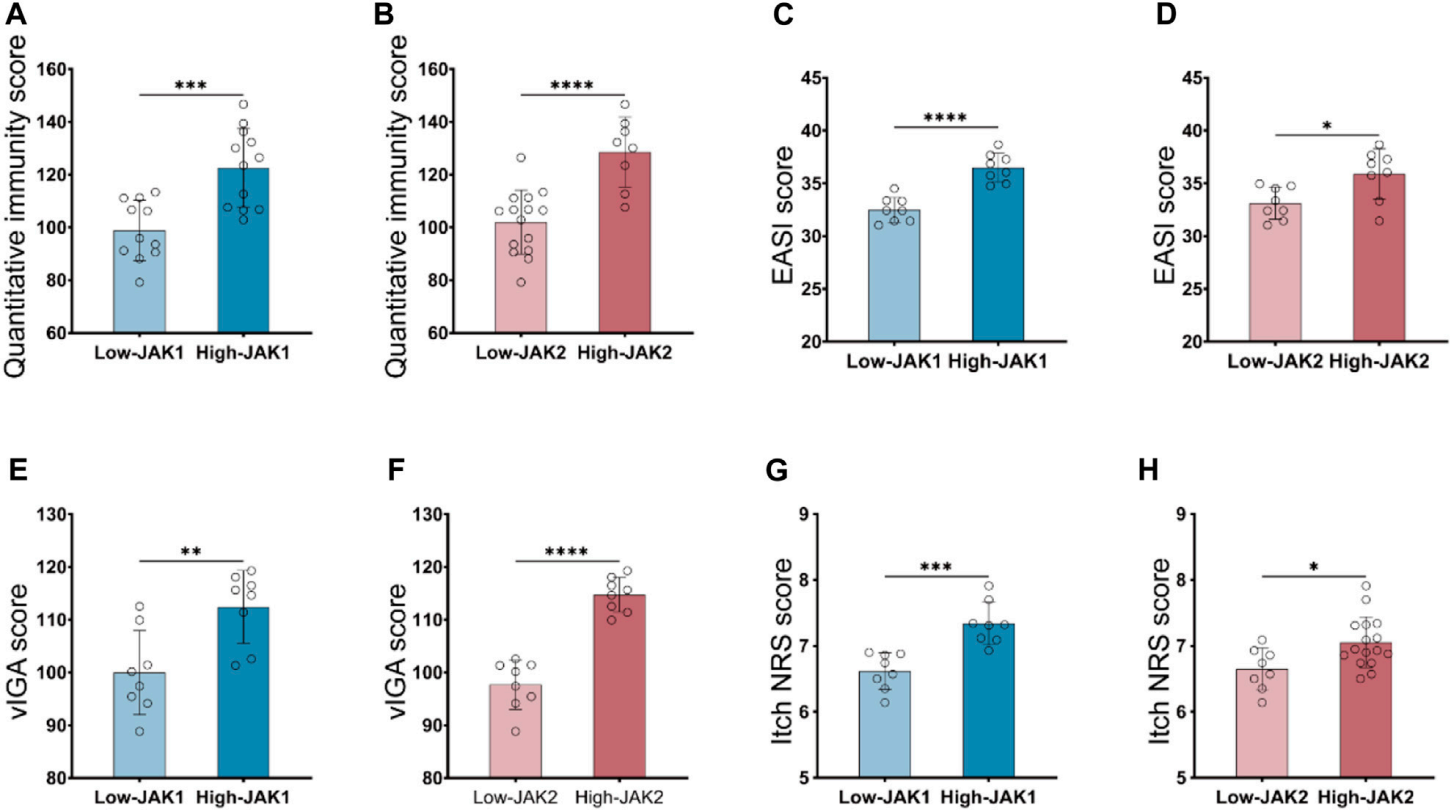
High JAK1/2 expression correlates with symptom severity in patients with moderate and severe AD. **(A, B)** The quantitative immunity score of initial moderate and severe AD patients with low-JAK1 group (*n* = 11) and high-JAK1 group (*n* = 12), or low-JAK2 (*n* = 15) group and high-JAK2 group (*n* = 8) using the quantitative immunity score system. **(C, D)** The eczema area and severity index score (EASI score) of moderate and severe AD patients with low-JAK1 group (*n* = 11) and high-JAK1 group (n = 12), or low-JAK2 group (*n* = 15) and high-JAK2 group (*n* = 8). **(E, F):** The validated investigator global assessment score (vIGA score) of moderate and severe AD patients with low-JAK1 group (n = 11) and high-JAK1 group (*n* = 12), or low-JAK2 group (*n* = 15) and high-JAK2 group (n = 8). **(G, H)** The itch numerical rating scale score (Itch NRS score) of moderate and severe AD patients with low-JAK1 group (*n* = 11) and high-JAK1 group (*n* = 12), or low-JAK2 group (n = 15) and high-JAK2 group (*n* = 8). Data are expressed as mean ± SD. *p < 0.05; **p < 0.01; ***p < 0.001; ****p < 0.0001.

JAK1/2 is exactly the target of the inhibitor baricitinib, which is a novel targeted drug for the treatment of autoimmune diseases, and has good therapeutic effects on diseases such as rheumatoid arthritis and AD. We then carried out a correlation analysis between the expression level of JAK1/2 and the clinical symptoms of AD patients. 23 AD patients were divided into low-JAK1/2 group (*n* = 11/15) and high-JAK1/2 (*n* = 12/8) group according to the expression level of JAK1 or JAK2 (Supplementary Figure S3). We found that the quantitative immunity score of AD patients with high JAK1/2 expression was significantly higher than that of AD patients with low JAK1/2 expression (Figures 2A, B). Moreover, the clinical symptom score of AD patients with high JAK1/2 expression, such as EASI Score (Hanifin et al., 2001; Schram et al., 2012), vIGA Score (Futamura et al., 2016), and Itch NRS score (Phan et al., 2012; Kimel et al., 2020) were also significantly higher than those of AD patients with low JAK1/2 expression (Figures 2C–H). These results suggest that JAK1 and JAK2 may be advantageous targets for AD immunotherapy.

### 3.3 Baricitinib suppressed AD skin lesions of mice and patients

**FIGURE 3:**
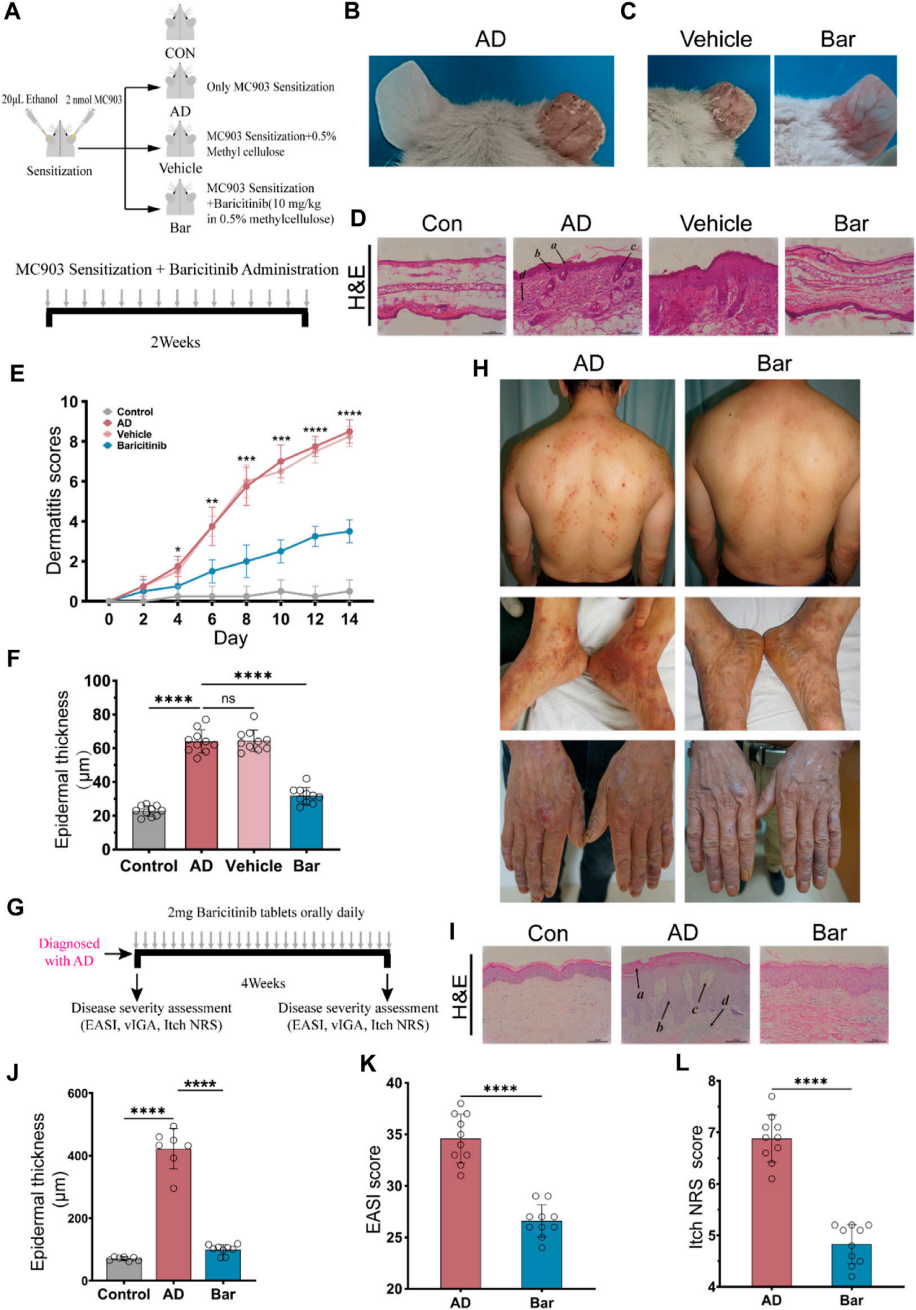
Baricitinib improves AD-like dermatitis symptoms in mice and patients. **(A)** Animal model construction: The AD mouse model was established by repeated stimulation on the right ear of the mouse with MC903 (2nmol/ear) for 14 days, mouse in the baricitinib group (*n* = 10) was simultaneously treated with baricitinib (1 mg/kg) by the oral administration. N = 10 per group. The treatments were carried out in triplicate. **(B)** Photographs of mice ear sections from the AD group on day 14. The right ear was coated with 2 nmol (20 μL) of MC903 (anhydrous ethanol as solvent), and the left ear was coated with an equal amount of anhydrous ethanol. **(C)** Photographs of mice ear sections from the Vehicle group and baricitinib group on day 14. **(D)** H&E staining of ear sections of mice from each group on day 14. Hyperkeratotic **(A)**, hyperplasia of the granular layer **(B)**, thickened dermis layer **(C)**, and a large number of inflammatory cells infiltrated in the dermis layer**(D)** were shown in the AD group. Original magnification ×200, Scale bar = 100 μm. **(E)** Dynamic changes in the score at indicated time points. Control group (n = 4) vs. AD group (*n* = 4), ***p < 0.001. AD group (*n* = 4) vs. baricitinib group, (*n* = 4), ***p < 0.001. **(F)** Epidermal thickness measured from H&E-stained ear sections of mice on day 14. **(G)** Medication procedures for moderate-to-severe AD patients. Patients were treated with once-daily 2 mg baricitinib tablets for 4 weeks. **(H)** Photograph of the skin on the back, feet, and hands of AD patients before and after baricitinib treatment (2 mg/day, 4 weeks). **(I)** H&E staining of skin from healthy control, initial moderate and severe AD patients, and initial moderate and severe AD patients after 4 weeks of baricitinib treatment. Hyperkeratotic **(A)**, hyperplasia of the granular layer **(B)**, vacuolated **(C)** with a large number of inflammatory cell infiltration in the dermis layer **(D)**. Original magnification ×200, Scale bar = 100 μm. **(J)** Epidermal thickness measured from H&E-stained skin of healthy control (*n* = 5), initial moderate and severe AD patients (*n* = 8), and initial moderate and severe AD patients after 4 weeks of baricitinib treatment (*n* = 7). **(K, L)**: The eczema area and severity index score (EASI score) and the itch numerical rating scale score (Itch NRS score) of initial moderate and severe AD patients (*n* = 23) before and after 4 weeks of baricitinib treatment. Data are expressed as mean ± SD. *p < 0.05; **p < 0.01; ***p < 0.001; ****p < 0.0001.

To verify whether inhibition of JAK1/2 can improve AD symptoms, we constructed an AD model of BALB/c mice induced by MC903 and treated mice with JAK1/2-targeting inhibitor baricitinib by gavage (Figure 3A). On the 14th day of the experiment, we observed the right ear stimulated with MC903 showed obvious AD-like skin lesions such as erythema, spotting hemorrhage, dryness, and crusting (Figure 3B). While the intervention of baricitinib, the AD-like ear skin lesions induced by MC903 were significantly improved (Figure 3C). We also found that the dermatitis scores of mice in the AD model group were significantly increased on the 14th day, and baricitinib treatment significantly reduced dermatitis scores in the baricitinib group (Figure 3E). Furthermore, we found that the skin lesions of mice in the AD model group showed hyperkeratotic (a), hyperplasia of the granular layer (b), thickened dermis layer (c), a large number of inflammatory cells infiltrated in the dermis layer (d) (Figure 3D) and the epidermis was thickened (Figure 3F), while these symptoms were significantly improved by baricitinib treatment. Likewise, we observed that the symptoms in AD patients (including redness, bleeding, and crusting) which were similar to the symptoms in mice were significantly improved by baricitinib treatment (Figure 3H). After staining the pathological tissue sections of AD patients, we found that the skin epidermis of AD patients was hyperkeratotic (a), hyperplasia of the granular layer (b), vacuolated (c) with a large number of inflammatory cell infiltration in the dermis layer (d) (Figure 3I). While these symptoms disappeared significantly after treatment with baricitinib for 4 weeks. Meanwhile, we analyzed the epidermal thickness of the skin in each group and found that baricitinib treatment significantly reduced the epidermal thickness of the skin lesions in AD patients (Figure 3J). Moreover, we evaluated the severity of dermatitis symptoms in AD patients before and after baricitinib treatment by using the EASI score and Itch NRS score system. We found that the EASI score and Itch NRS score of AD patients were significantly lower after baricitinib treatment (Figures 3K, L). These results suggested that baricitinib had improved the dermatitis symptoms of AD patients and mice.

### 3.4 Treatment with baricitinib reduces T-cell activation of moderate and severe AD patients

**FIGURE 4:**
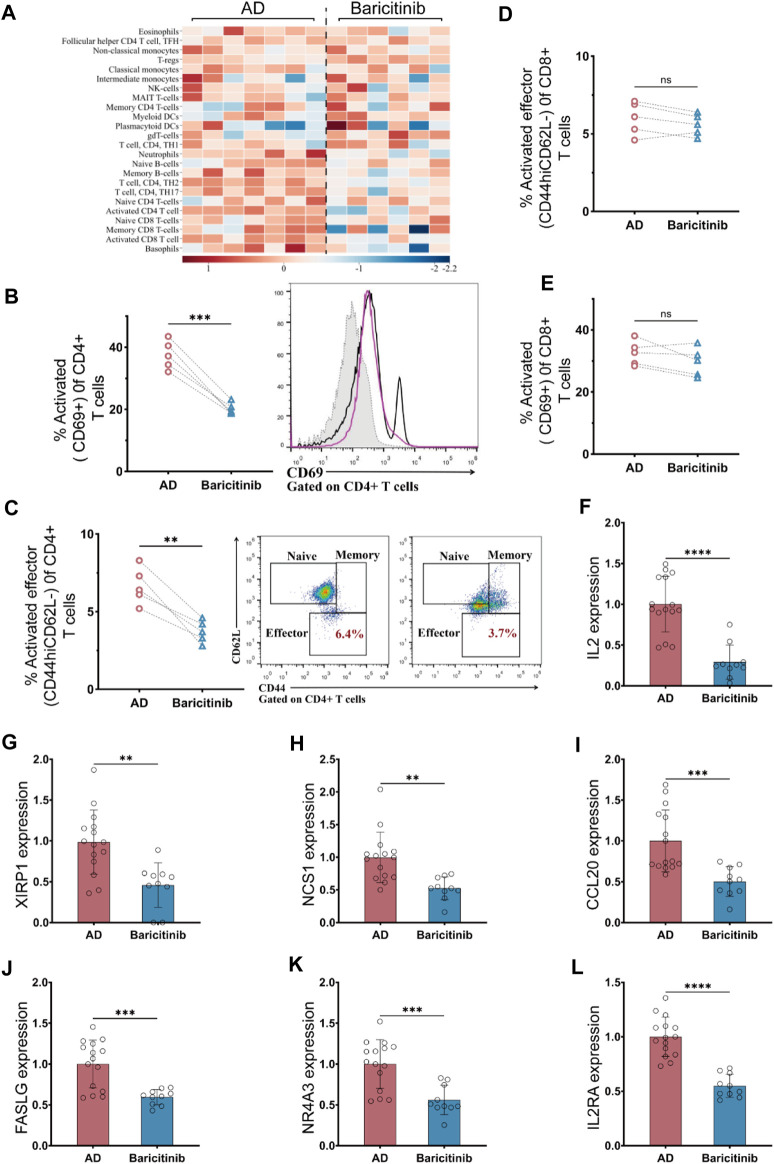
Baricitinib inhibits T-cell activation in patients with moderate and severe AD. **(A)** The Immunological function of peripheral blood PBMC subpopulations in moderate and severe AD patients before and after baricitinib treatment was assessed using the Immune Cell Function Scoring System, and heatmaps show the fold change about the level of immunological function of multiple inflammatory cells before and after baricitinib treatment. **(B, E)**The activated levels of T cells in the peripheral blood of AD patients before and after baricitinib treatment were detected by FCM assay. The plots show CD69 staining gated on live CD4^+^ **(B)** or CD8^+^ **(E)** T cells, and the graphs show the percentage of activated effector subsets (CD69^+^) among CD4^+^ or CD8^+^ T cells. **(C, D)** The activated levels of T cells in the peripheral blood of AD patients before and after baricitinib treatment were detected by FCM assay. The plots show CD62L and CD44 staining gated on live CD4^+^ **(C)** or CD8^+^ **(D)** T cells, and the graphs show the percentage of activated effector subsets (CD44^+^CD62L−) among CD4^+^ or CD8^+^ T cells. **(F–L)**: The expression levels of different cytokines (IL2, XIRP1 NCS1, CCL20, FASLG, IL2RA, NR4A3) in the peripheral blood of AD patients before and after baricitinib treatment were detected by real-time PCR.

To investigate which groups of immune cells were affected by baricitinib in AD patients (*n* = 23), peripheral blood PBMC was collected from moderate and severe AD patients before and after baricitinib treatment and assessed the functional levels of immune cell subsets. We found that baricitinib treatment suppressed the functional levels of inflammatory cells, especially T-cell subsets, including activated CD8^+^ T-cells, memory CD8^+^ T-cells, CD4^+^ TH2 cells, and CD4^+^ TH17 by RNA-Seq (Figure 4A).

Therefore, we further analyzed the activation levels of CD4^+^ T cells and CD8 T-cell subpopulations in the peripheral blood of moderate and severe AD patients (*n* = 5) before and after baricitinib treatment by FCM assay. The proportion of activated (CD69^+^) CD4^+^ T cell subset and activated Effector (CD44hiCD62L-) CD4^+^ T cell subset was significantly decreased after baricitinib treatment (Figures 4 B, C). And the proportion of activated (CD69^+^) CD8 T cells and activated Effector (CD44hiCD62L-) CD8 T cell population was slightly decreased (Figures 4 D, E). Moreover, we found that the expression levels of T-cell activating factors (IL2, XIRP1 NCS1, CCL20, FASLG, IL2RA, NR4A3) in peripheral blood of moderate and severe AD patients were significantly downregulated after baricitinib treatment (Figures 4F–L). These results indicate that baricitinib treatment inhibits T-cell activation.

### 3.5 Baricitinib downregulates the activation of CD4^+^ TH2 cells and its effector molecules in moderate and severe AD

**FIGURE 5:**
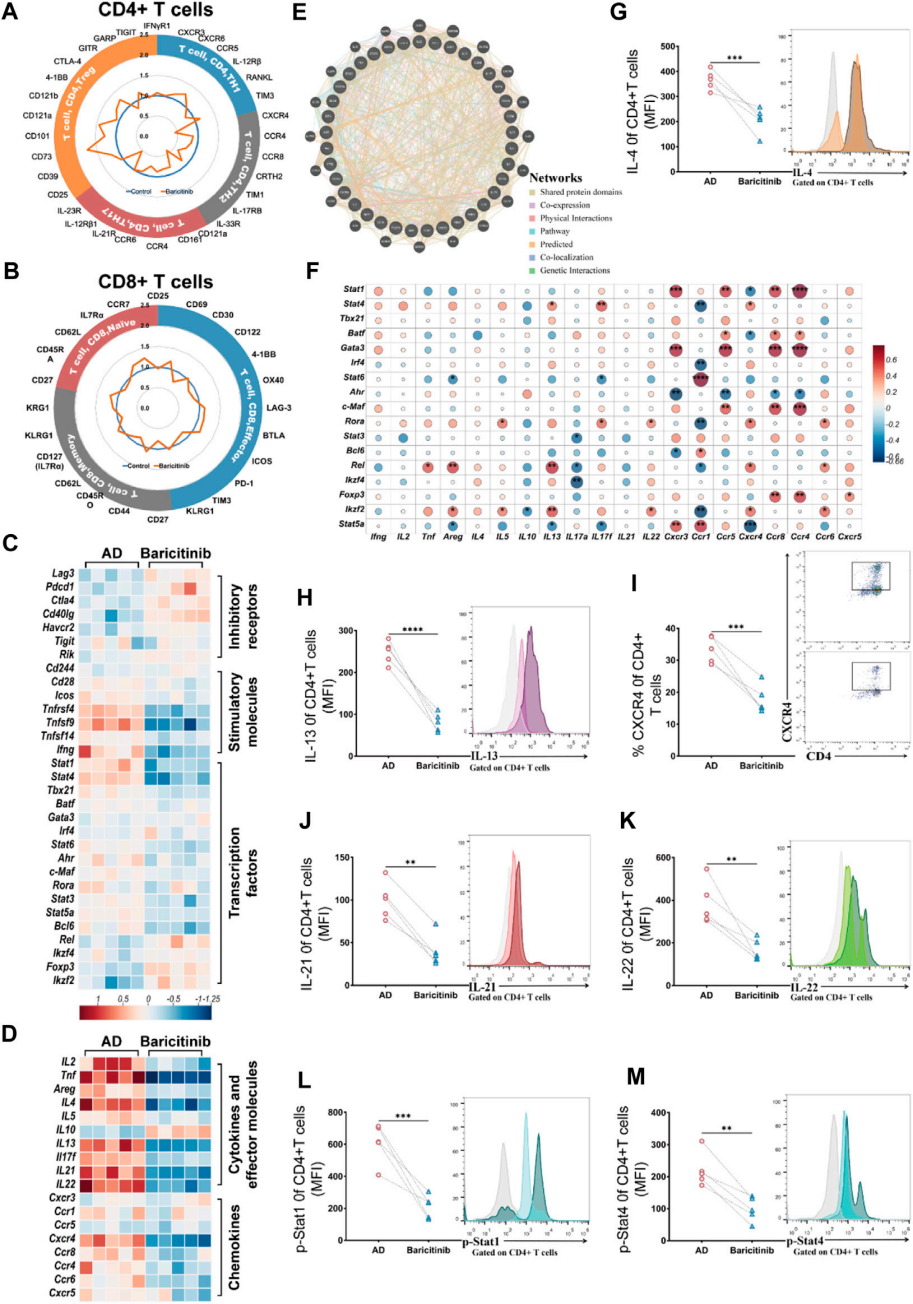
Baricitinib inhibits activation of TH2 cell subpopulation and its effectors molecules in AD patients. **(A, B)** Comparison of the fold changes in the expression levels of uniquely expressed genes in CD4^+^ T cells (including CD4^+^ TH1, CD4^+^ TH17, CD4^+^TH2, and CD4^+^ T Treg subsets) or CD8^+^ T cells (including CD8^+^ Effector, CD8^+^ Memory, and CD8^+^Naive subsets) from lymphocytes of AD patients (n = 23) before and after baricitinib treatment. A circle diagram of unique gene expression revealed some interesting differences in CD4^+^ TH17 and CD4^+^ TH2 subsets. **(C, D)** Comparison of transcriptional profiles of inhibitory receptors genes, stimulatory molecules genes, transcription factors genes, cytokines genes, effector molecules genes and chemokines genes in peripheral blood CD4^+^ T cell subsets from AD patients (*n* = 23) before and after baricitinib treatment. Heat map showing differentially expressed genes with fold change differences. **(E)** The transcription factors genes, Cytokines genes, effector molecules genes and Chemokines genes of CD4^+^ T cell subsets (Shared protein domains: 30.07%, co-expression: 28.96%, physical interactions: 14.46%, pathway: 11.18%, predicted: 10.24%, co-localization: 4.33%, genetic interactions: 0.75%) that interacted each other were identified through the GeneMANIA database (Version: 3.6.0; network weighting: automatically selected weighting method). **(F)** Matrix correlation analysis of Transcription factors and Cytokines, effector molecules Chemokines gene expression levels in peripheral blood CD4^+^ T cell subsets with fold change differences at *p* < 0.05. **(G–K):** The secretion levels of multiple cytokines (IL-4, IL-13, CXCR4, IL-21, IL-22) in the peripheral blood of AD patients (n = 23) before and after baricitinib treatment were detected by Flow CytoMetry. **(L, M)**: The phosphorylation levels of Stat1 and Stat4 in the peripheral blood of AD patients (n = 23) before and after baricitinib treatment were detected by Flow CytoMetry.

To investigate the inhibitory effect of baricitinib on T-cell activation, we next analyzed the changes in maker genes of CD4^+^ T-cell subsets and CD8^+^ T-cell subsets in peripheral blood of moderate and severe AD patients (*n* = 23) before and after baricitinib treatment by RNA-seq. The results showed that baricitinib significantly suppressed the expression of marker genes in CD4^+^TH2 and CD4^+^TH17 cell subsets and upregulated the expression of marker genes in CD4^+^Treg cell subsets (Figure 5A). In contrast, there was no significant change in marker gene expression in CD8^+^ T cell subsets before and after baricitinib treatment compared to CD4^+^ T cell subsets (Figure 5B), suggesting that baricitinib functioned mainly by suppressing CD4^+^ T cell subsets. To confirm the inhibitory effect of baricitinib on CD4^+^ T cell subsets, we next analyzed the expression levels of inhibitory receptors, stimulatory molecules and transcription factors of CD4^+^ T cell subsets in peripheral blood of AD patients before and after baricitinib treatment. We found that baricitinib upregulated the expression of CD4^+^ T cell inhibitory receptors (such as Pdcd1, Cd40lg) and suppressed the expression of CD4^+^ T cell stimulatory molecules (Tnfrsf4, Tnfsf9, Ifng) (Figure 5C). Meanwhile, baricitinib downregulated the expression levels of Transcription factors (Stat1, Stat4, Stat3, Stat5a, Bcl6) (Figure 5C). Similar to the expression levels of transcription factors, baricitinib also downregulated the CD4^+^ T cell subsets cytokines and effector molecules (Tnf, Il4, Il13, Il21, Il22) and chemokines (Cxcr4, Ccr4, Ccr6) expression levels (Figure 5D). In addition, we observed that Baricitinib had no significant effect on CD8^+^ T cell subsets among inhibitory receptors, stimulatory molecules, transcription factors, cytokines, and effector molecules (Supplementary Figure S4). To further explore the mechanism by which baricitinib suppressed CD4^+^ T cell immunity, the transcription factors genes, Cytokines genes, effector molecules genes and Chemokines genes of CD4^+^ T cell subsets were analyzed by the GeneMANIA database. We found that the CD4^+^ T cell subsets of transcription factors genes (such as Stat4, Stat3, Stat5a) could regulate Cytokines genes, effector molecules genes and Chemokines genes through multiple pathways including Co-expression, Physical Interactions, and Pathways (Figure 5E). We also found that Transcription factors (Stat1, Stat4, Stat6, Gata3, c-MAF) were significantly correlated with Cytokines, effector molecules and Chemokines by performing matrix correlation analysis of gene expression levels of both groups (*p* < 0.05) (Figure 5F). Moreover, we further confirmed that baricitinib inhibited the expression of effector molecules (IL-4, IL-13, CXCR4, IL-21, IL-22) (Figures 5G–K) and inhibits the phosphorylation of transcription factors (such as Stat1 and Stat4) in CD4^+^ T cell of AD moderate and severe patients after baricitinib treatment by flow cytometry analysis (Figures 5L, M). These results suggest that baricitinib targets the transcription factors (such as Stat4, Stat3, Stat5a) of CD4^+^T cells to downregulate IL-4, IL-13, CXCR4, IL-21, IL-22 effector molecules through multiple pathways to improve AD.

### 3.6 Baricitinib may inhibit T cell-mediated inflammatory responses through MAPK, mTOR and PI3K/Akt signaling pathway via JAK-STAT signaling

**FIGURE 6:**
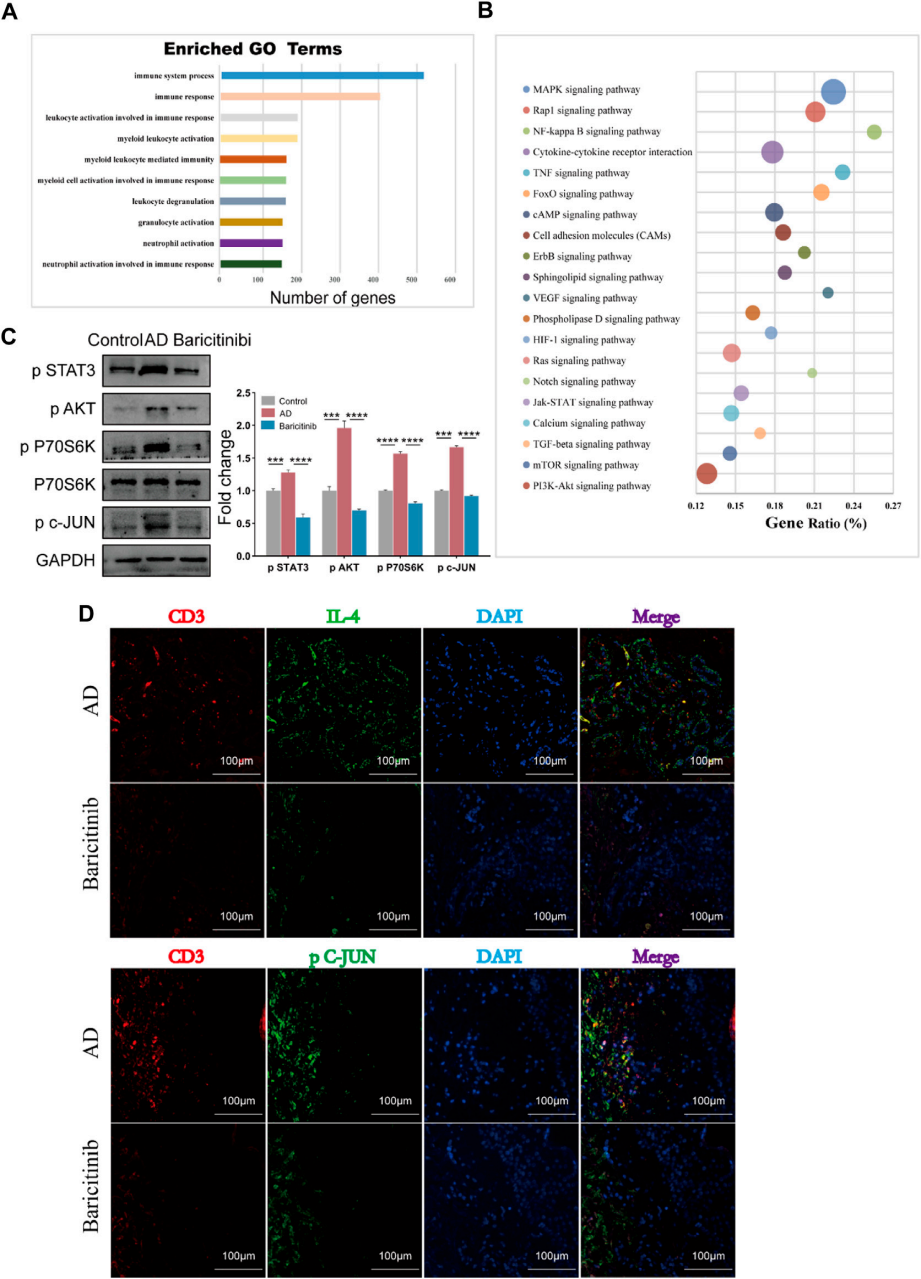
Baricitinib may inhibit T cell-mediated inflammatory responses through MAPK, mTOR and PI3K/Akt signaling pathway via JAK-STAT signaling. **(A)** The enrichment analysis of GO analysis showed that the differentially expressed genes after baricitinib treatment. The functional enrichment of these genes mainly involved in neutrophil activation involved in immune response, neutrophil activation, granulocyte activation, leukocyte degranulation, myeloid cell activation involved in immune response, myeloid leukocyte mediated immunity, myeloid leukocyte activation, leukocyte activation involved in immune response, immune response and immune system process (*p* < 0.05). **(B)** The enrichment analysis of KEGG pathway analysis showed the top 20 pathways about differentially expressed genes after baricitinib treatment including MAPK signaling pathway, Jak-STAT signaling pathway, mTOR signaling pathway, PI3K-Akt signaling pathway (*p* < 0.05). **(C)** The expression levels of p STAT3, p P70 S6K, p AKT and p c-JUN in the ear of AD mice induced by MC903 were evaluated by Western blotting (n = 4 per group). The gels were run under the same experimental conditions. *p < 0.05; **p < 0.01; ***p < 0.001; ****p < 0.0001. **(D)** Immunofluorescence staining of skin lesion tissue of AD patients before and after baricitinib treatment. The circles show the staining of CD3, IL4, and P c-JUN in the AD group. Original magnification ×200, Scale bar = 100 μm.

To analyze further mechanisms underlying the inhibition of T cell activation and production of effectors such as IL-4, IL-13, CXCR4, IL-21, and IL-22 by baricitinib, we next used transcriptome sequencing of AD patients’ T cells. Go analysis showed that the functional enrichment (*n* = 293) of these differential genes main involved in lymphocyte activation and immune response (Figure 6A), suggesting that baricitinib treatment inhibits the production of inflammatory stimulating factors. KEGG analyses revealed that there are 78 pathways with differences, the top twenty pathways included MAPK, Jak-STAT, mTOR, and PI3K-Akt signaling pathways (Figure 6B). Next, we constructed an MC903-induced AD model in the BALB/c mouse ear section and treated the mice by gavage with or without the JAK1/2-targeting inhibitor baricitinib. Western blotting of the ear lesions of the AD mouse model showed that baricitinib treatment effectively downregulated the p-STAT3, p-AKT, and p-P70S60K, compared with the non-treatment AD group (Figure 6C). This implies that baricitinib inhibits Jak-STAT signaling while suppressing its downstream PI3K/Akt and mTOR pathways. Baricitinib also downregulated the level of phosphorylated c-JUN (p c-JUN) (Figure 6C), suggesting that baricitinib also significantly inhibits the MAPK pathway. In addition, fluorescence staining of tissue sections from clinical AD patients also confirmed that baricitinib treatment effectively downregulated the secretion of IL4 and the phosphorylation level of c-JUN (Figure 6D). We further explored whether the inhibition of the MAPK pathway, PI3K-Akt, and mTOR pathway was produced by baricitinib’s inhibition of Jak-STAT signaling.

### 3.7 Baricitinib may improve AD through MAPK pathway, PI3K/Akt pathway and mTOR pathway via JAK-STAT signaling

**FIGURE 7:**
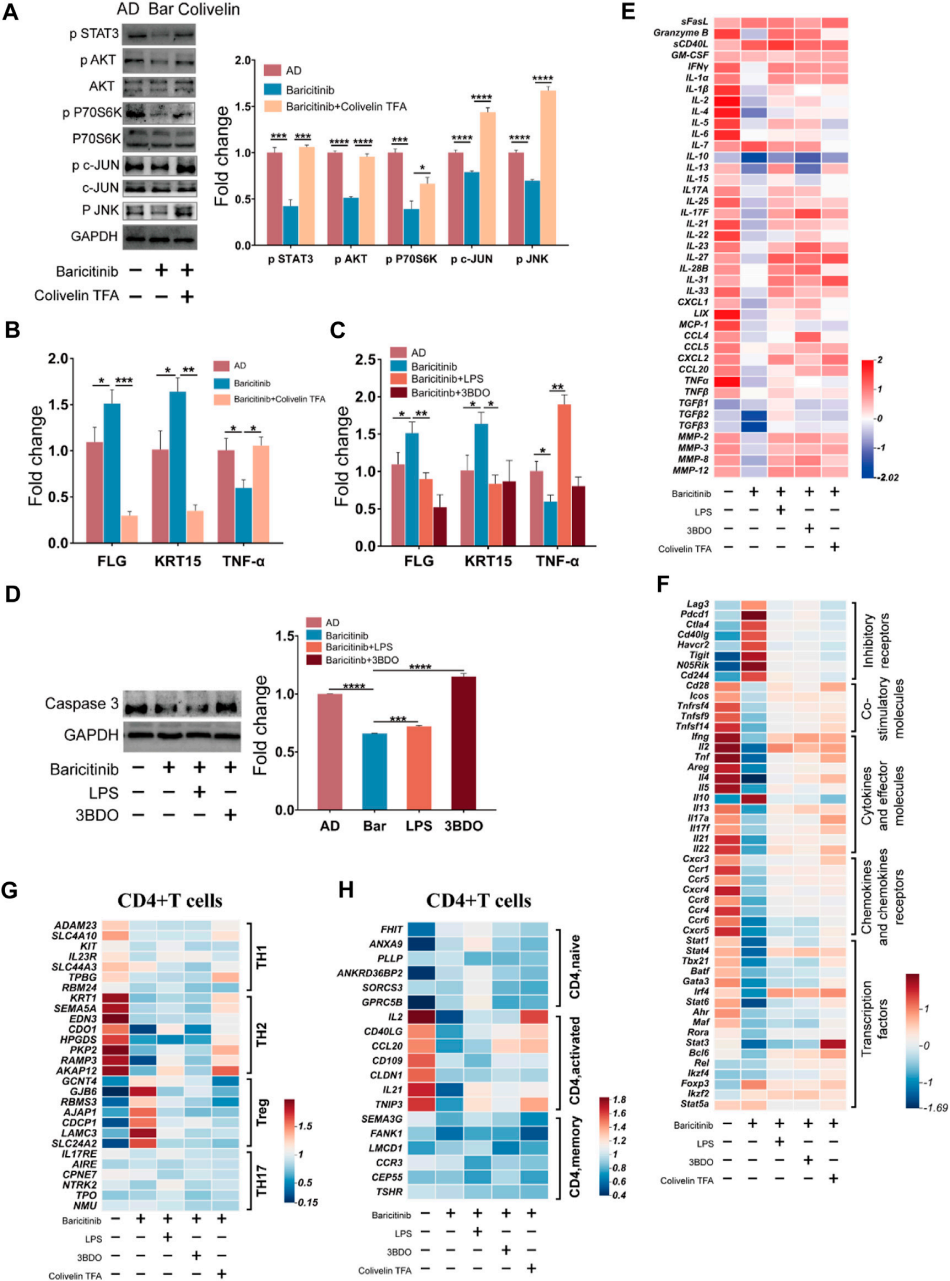
Baricitinib may improve AD through MAPK pathway, PI3K/Akt pathway and mTOR pathway via JAK-STAT signaling. **(A)** The expression levels of c-JUN, AKT, P70S60K, phospho c-JUN, phospho JNK, phospho AKT, phospho P70S60K, phospho STAT3 in PBMCs of AD patients (*n* = 4 per group) were evaluated by Western blotting after 24 h co-culture with lesion tissue, baricitinib (55.4 nM), and Colivelin TFA (20μM; the activator of the JAK-STAT pathway) (*p* < 0.05). The gels were run under the same experimental conditions. **(B)** The transcription levels of mRNA of FLG, KRT15, and TNF-α in lesion tissue co-cultured with PBMCs of AD patients after intervening with baricitinib (55.4 nM) and Colivelin TFA (20 μM) for 24 h were evaluated by Real-time Quantitative PCR (*p* < 0.05). **(C)** The transcription levels of mRNA of FLG, KRT15, and TNF-α in lesion tissue co-cultured with PBMCs of AD patients after intervening with baricitinib (55.4 nM), LPS (10 μg/ml) and 3DDO (60 μM) for 24 h were evaluated by Real-time Quantitative PCR (*p* < 0.05). LPS is the activator of the MAPK pathway and the PI3K-AKT pathway and 3BDO is the activator of the mTOR pathway. **(D)** The expression levels of caspase3 in PBMCs of AD patients (*n* = 4 per group) were evaluated by Western blotting after 24 h co-culture with lesion tissue, baricitinib (55.4 nM), LPS (10 μg/ml) and 3DDO (60 μM) (*p* < 0.05). The gels were run under the same experimental conditions. **(E)** The expression level of cytokines in the culture medium of the co-culture system was measured by ELISA assay. **(F)** Comparison of transcriptional profiles of inhibitory receptors genes, co-stimulatory molecules genes, cytokines and effector molecules genes, chemokines and chemokines receptors genes, and transcription factors genes in lymphocytes of AD patients after 24 h co-culture with lesion tissue, baricitinib (55.4 nM), LPS (10 μg/ml), 3DDO (60 μM) and Colivelin TFA (20 μM). Heat map showing differentially expressed genes with fold change differences at *p* < 0.05. **(G, H)** Comparison of the fold changes in the expression levels of uniquely expressed genes in CD4^+^ T cells (including CD4^+^ TH1, CD4^+^TH2, CD4^+^ Treg, and CD4^+^ TH17 subsets) or activation genes in CD4^+^ T cells (including CD4^+^naive, CD4^+^activated, and CD4^+^memory subsets) from lymphocytes of AD patients after 24 h co-culture with lesion tissue, baricitinib (55.4 nM), LPS(10 μg/ml), 3DDO (60 μM) and Colivelin TFA ((20 μM). Heat map showing differentially expressed genes with fold change in CD4^+^ Treg CD4^+^TH2 subsets and CD4^+^activated subsets in baricitinib groups.

To verify the effect of baricitinib on the MAPK pathway, PI3K-Akt pathway, and mTOR pathway by inhibiting Jak-STAT signaling in the treatment of AD, T cells and skin lesion tissues from moderate and severe AD patients were placed in the upper and lower chambers of Transwell respectively for mixed culture. Western blotting assays of T cells revealed that baricitinib reduced the phosphorylation levels of c-JUN (MAPK pathway protein) along with JNK, AKT (PI3K/Akt pathway protein), and P70S60K (mTOR pathway protein) (Figure 7A), while STAT3 agonists (Colivelin TFA) treatment relieved the inhibitory effect of baricitinib on the phosphorylation of c-JUN, AKT, and P70S60K, and the phosphorylation levels were significantly increased (Figure 7A). All data above suggest that baricitinib may have a regulatory effect on the JNK branch of the MAPK pathway, the PI3K-AKT pathway, and the mTOR pathway by inhibiting the JAK-STAT signaling in T cells. Meanwhile, we performed qPCR analysis on mixed cultured skin lesion tissues of AD patients to detect the transcript levels of skin barrier function genes FLG, KRT15, and inflammatory factor TNF-α. The results showed that FLG and KRT15 transcript levels were significantly higher and TNF-α transcript levels were significantly lower in the baricitinib group compared to the AD group (*p* < 0.05), while FLG and KRT15 transcript levels were lower and TNF-α transcript levels were higher after intervention with STAT3 agonists (Figure 7B). Therefore, we speculate that baricitinib may inhibit T cells function through MAPK, PI3K/AKT, and mTOR pathways protecting skin lesions in AD disease. Next, we selected the agonists of the three pathways to explore whether the activation of the three pathways would inhibit the protective effects of baricitinib on skin lesions of AD patients. As speculated, LPS and 3BDO intervention partially relieved the protective effect of baricitinib on mixed cultured skin lesion tissues, the expression of TNF-α (Figure 7C) and apoptosis protein caspase3 (Figure 7D) were significantly increased and the expression of FLG and KRT15 were significantly decreased in co-cultured skin lesion tissues (Figure 7C).

We further detected the cytokines in the mixed culture medium and found that baricitinib inhibited the secretion of TH2 inflammatory cytokines such as IL2, IL4, IL21, and IL22 by lymphocytes, while the intervention with LPS, 3BDO, and Colivelin TFA relieved baricitinib’s inhibitory effect on the secretion of these inflammatory cytokines (Figure 7E). We also analyzed the unique expression genes of CD4^+^ T cell subsets after mixed culture and found that baricitinib inhibited the expression of the marker gene in TH2 cell subsets and CD4^+^ T cell activation factors (such as IL2, CD40LG, CCL20, CD109, CLDN1, IL21, and TNIP3) and upregulated the expression of the marker gene in the Treg cell subset (Figures 7G, H). Moreover, we found that baricitinib upregulated the expression of inhibitory receptors (such as Lag3, Pdcd1, Ctla4, Cd40lg, Havcr2, Tigit, and Cd244), and inhibited the expression of the co-stimulatory molecules (such as Cd28, Icos, Tnfrsf4, Tnfsf9, and TNFSF14), the effector molecules (such as Ifng, Il2, Tnf, Areg, Il4, Il5, Il10, Il13, Il17a, Il17f, Il21, Il22) and the chemokines and chemokines receptors (such as Cxcr3, Ccr1, Ccr5, Cxcr4, Ccr8, Ccr4, Ccr6, Cxcr5). While the intervention of LPS, 3BDO, and Colivelin TFA relieved the regulatory effect of baricitinib on CD4^+^ T cell subsets (Figure 7F), suggesting that baricitinib can inhibit the activation of CD4^+^ T cells and the secretion of TH2 cytokines. Besides, baricitinib downregulated the expression of transcription factors (such as Stat1, Stat4, Tbx21, Batf, Gata3, Stat6, Stat3, and Bcl6) in CD4^+^ T cell subsets, while the expression of these transcription factors was upregulated to the similar levels in AD group after the intervention of Colivelin TFA (Figure 7F). Those results indicate that this skin lesion protective effect of baricitinib may be to inhibit the TH2 differentiation and function of CD4^+^T by inhibiting the JAK-STAT signal to downregulate MAPK, PI3K-AKT, and mTOR pathways.

## 4 Discussion

Atopic dermatitis is a chronic inflammatory skin disease with pruritus as a clinical symptom and prone to recurrence, the etiology of which is not fully understood, but there are many studies have shown the possible mechanisms of AD. Various susceptibility genes, environmental factors, skin barrier defects, and immune responses play a dominant role in AD (Langan et al., 2020). Epidermal keratinocytes, which form a functional skin barrier, provide the first line of defense against pathogen invasion, irritants, and allergens. Indeed, multiple studies have shown that AD is an immune disorder closely associated with elevated production of IgE and secretion of T helper (Th)2 cytokines (Hamilton et al., 2014). There is evidence that AD patients are in a state of hyper immunity, and activated keratinocytes at the lesion induce a large number of Th2 cells into the inflammatory lesion, leading to an imbalance of TH1 and TH2 cells. Th2 cells enter the inflammatory lesions and induce increased expression of Th2 cytokines, including IL-4, IL-5, and IL-13, as well as the conversion of IgE classes in B cells (Hamid et al., 1994; Taha et al., 2000; Hamilton et al., 2014), which accounts for the increased IgE levels, causing thickening, inflammation, and pruritus of the epidermis, along with downregulation of skin barrier proteins, such as filaggrin (FLG) and KRT15 which are essential for maintaining skin barrier function (Drislane and Irvine, 2020; Hoober and Eggink, 2022; Ievlev et al., 2023). The results of flow cytometry analysis of the patient’s peripheral blood PBMC similarly confirmed that AD is an immune disorder characterized by abnormal activation of TH2 cell subsets and the expression of TH2-type cytokines such as IL-4 and IL-13 is increased in AD patients. We also demonstrated that B inhibited the activation of TH2 cells and the secretion of its effector molecules, and upregulated the expression of FLG and KRT15 to improve AD.

The current common treatment for AD is the use of steroids including topical corticosteroids (e.g., glucocorticoids), topical calcineurin inhibitors (e.g., tacrolimus and pimecrolimus), or both, for anti-inflammatory treatment of visible skin lesions (Wollenberg et al., 2016). Although these topical treatments can alleviate AD symptoms and reduce inflammation, they are associated with side effects of long-term use. These side effects include local skin atrophy, stripe formation skin atrophy from topical corticosteroids, and tingling from topical calcineurin inhibitors. Therefore, improving immune system disorders, inhibiting abnormal TH2 activation, and reducing TH2-type cytokine secretion in AD patients may be a better therapeutic strategy than the common anti-inflammatory treatment for skin lesions. Targeted agent therapies for AD have gained wide recognition in recent years. Many pharmaceutical companies are currently developing drugs for different AD therapeutic targets, such as IL-4, IL-5, IL-13, TSLP, IL-12, IL-23, IL-31, IL-17, and IgE (Beck et al., 2014; Romano et al., 2015; Khattri et al., 2017; Ruzicka and Mihara, 2017; Kabashima et al., 2018). In our study, after analyzing the transcript levels of 53 immunotherapeutic targets in peripheral blood immune cells of patients, we found that JAK1/2 are 2 of the 3 immunotherapeutic targets with the highest rise in transcript levels and therefore may be potential targets for the treatment of AD. We treated first-time AD patients with baricitinib, which exactly inhibits the target JAK1/2, for 4 weeks and found a significant reduction in the patient’s dermatitis symptoms and pruritus level. Baricitinib had a similar effect on dermatitis symptoms in AD mice. AD is an autoimmune skin disease caused by abnormal activation of TH2 cell subsets and elevated secretion of Th2 cytokines such as IL-4. Our results show that baricitinib inhibits the activation of TH2 cell subsets in the peripheral blood of AD patients, meanwhile downregulating the secretion levels of T cell effector molecules (including IL-4, IL-13, CXCR4, IL-21, IL-22). Therefore, treatment with baricitinib may be a better option compared to traditional AD treatment.

Several clinical trials have shown that baricitinib reduces eczema area and severity index (EASI-50) at 12 weeks while rapidly improving pruritus and sleep deprivation and enhancing the quality of life in adult patients with moderate-to-severe AD (Guttman-Yassky et al., 2019). There is evidence showing that baricitinib may improve dermatitis symptoms by suppressing IL-17 and IL-22 levels in the peripheral blood of AD patients through the JAK-STAT signaling pathway (Markham, 2017). Indeed, a number of researches have confirmed the anti-inflammatory effects of baricitinib in a wide range of diseases. Baricitinib’s inhibition on JAK-STAT signaling alleviated the progression of autoimmune diseases (e.g., systemic lupus erythematosus, Sjögren’s syndrome), inflammatory diseases (e.g., Hemorrhagic shock, Pseudoxanthoma elasticum (PXE)), and hypersensitivity reactions (e.g., eosinophilic cellulitis (EC)) (Aota et al., 2021; Lee et al., 2021; Lindenkamp et al., 2023; Morot et al., 2023; Patel et al., 2023). However, less has been reported on the mechanism by which baricitinib improves AD. JAK-STAT signaling pathway activates multiple downstream signals, and many cytokines involved in the pathogenesis of inflammatory and autoimmune diseases transduce intracellular signals through the JAK-STAT signaling pathway. AKT -PI3K pathway is an upstream activation pathway of mTOR, which is involved in biological processes such as gene transcription, protein translation, and ribosome synthesis, and plays an extremely important role in cell growth, apoptosis, autophagy, and metabolism. It has been shown that JAK-STAT signaling activates the AKT-PI3K, and mTOR signaling pathways (Zhang et al., 2022; Kumar et al., 2023). Our experimental results confirm that inhibition of the JAK-STAT signaling pathway with baricitinib does inhibit the activation of AKT-PI3K, and mTOR signaling pathways. The MAPK signaling pathway is an important signaling system for eukaryotic cells to transduce extracellular signals into the cell to elicit cellular responses and plays a role in cell proliferation, differentiation, apoptosis, stress, inflammation, and immune responses. It has been confirmed that JAK-STAT signaling can also activate the classical Ras-Raf-MEK-ERK pathway of the MAPK signaling pathway (Zhang et al., 2022). Furthermore, more evidence suggests that MAPK pathway activation may be closely related to the skin barrier and inflammation (Ni et al., 2022). The results of our study also found that baricitinib blocked Th2 cell subpopulation activation and Th2-type cytokines secretion by inhibiting JAK-STAT signaling. Moreover, we found that inhibition of the JAK-STAT signaling pathway by baricitinib was followed by inhibition of JNK bypass activation in the MAPK signaling pathway, which attenuated inflammatory responses and improved skin barrier better in AD patients. Whereas few previous reports on JAK-STAT signaling to regulate JNK bypass was available.

In our study, we further observed that Baricitinib upregulated the expression of the maker gene in CD4^+^ Treg cells (Figure 5A). Regulatory T cells (Treg) are a category of T cell subset with significant immunosuppressive effects, characterized by the expression of Foxp3, CD25, and CD4 as their cellular phenotype (Dikiy and Rudensky, 2023). Its absence or dysfunction usually contributes to the development of autoimmune diseases (Miyara et al., 2014; Wing et al., 2019). CD4^+^ Treg cells were important in the disease processes of atopic dermatitis too. A lot of researches suggest that there exists an immune imbalance in Th17 and Treg cells in AD patients (Ma et al., 2014), thus therapeutic strategy that expand Treg cells or enhance their inhibitory function are valuable for AD patients (Ou et al., 2004; Kim et al., 2018; Jia et al., 2021; Ahn et al., 2022). Similar to other studies, we observed upregulation of Treg cells in our study during Baricitinib treatment of AD. It suggests a potential possibility that Baricitinib may have a dual effect of reducing TH2 and increasing T-reg.

We observed that Baricitinib was effective in relieving symptoms in patients with moderate to severe AD in our study. However, one limitation of our study was the sample size, which limited us to determine the efficacy of Baricitinib in multiple age groups of AD patients, particularly adolescents and infants. Another restriction was the clinical trial design. There were multiple limitations on the clinical trial design including non-placebo controlled, short term follow-up period, single dose because of the small sample size of patients.

Although we demonstrated the beneficial effects of Baricitinib on mixed cells of skin lesion by co-culture system, we have not further investigated which type of epithelial cells were altered due to the difficulty of obtaining skin lesion tissues and sorting epithelial cells from AD patients. However, we believe that the effect of Baricitinib on different epithelial cells deserves further investigation.

## 5 Conclusion

**FIGURE 8:**
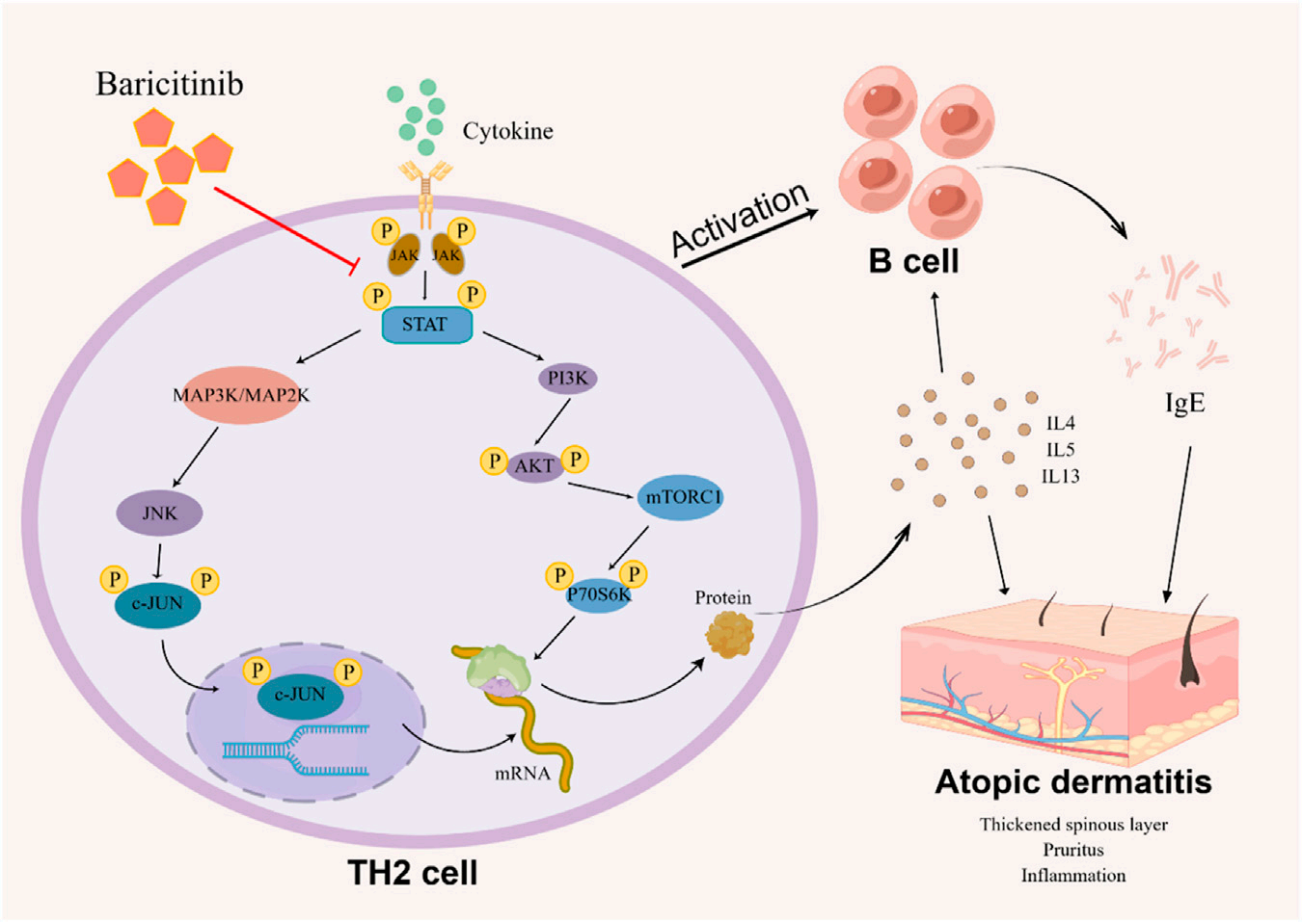
The image (by Figdraw) shows the possible mechanism that how baricitinib improves AD: Baricitinib inhibits TH2 cells activation by suppressing JAK-STAT signaling to downregulate the MAPK pathway, PI3K/Akt pathway, and mTOR pathway, decreasing B cell activation and reducing TH2 cytokine and IgE expression.

Taken together, our study confirms that baricitinib can improve the disturbed immune system and clinical symptoms in AD patients by inhibiting Th2 cell subpopulation activation and Th2-type cytokines secretion (Figure 8). Our experiments also suggest that the ameliorating effect of baricitinib on AD may be achieved by inhibition of three pathways, MAPK, mTOR, and AKT-PI3K through JAK-STAT signaling. We also newly identified that the JNK bypass in the MAPK signaling pathway may be a downstream pathway regulated by JAK-STAT signaling.

## Figures and Tables

**Table:** 

**AD**	atopic dermatitis
**TSLP**	thymic stromal lymphopoietin
**Bar**	baricitinib
**EASI**	eczema area and severity index
**IGA**	investigator´s global assessment
**NRS**	numeric rating scales
**FLG**	filaggrin
**KRT**	keratin
**JAK**	janus kinase
**STAT**	signal transducers and activators of transcription
**LPS**	lipopolysaccharide
**PBMC**	peripheral blood mononuclear cell
**TLR9**	Toll-like receptor 9
**CCR4**	C-C chemokine receptor type 4
**CCL17**	chemoattractant cytokine ligand 17
**PDCD10**	programmed cell death 10
**COX2**	cyclooxygenase-2
**MAPK**	mitogen-activated protein kinase
**mTOR**	mammalian target of rapamycin
**PI3K**	Phosphoinositide 3-kinase
**AKT**	Protein Kinase B
**c-JUN**	c-Jun N-terminal kinase
**JNK**	Jun N-terminal Kinase
**TH**	helper T cell
**IgE**	immunoglobulin E
**RA**	rheumatoid arthritis
**FCM**	Flow Cytometry
**TNF**	tumor necrosis factor
**Cxcr4**	C-X-C chemokine receptor type 4
**XIRP1**	Xin Actin Binding Repeat Containing 1
**NCS1**	neuronal calcium sensor-1
**FASLG**	factor-related apoptosis ligand

## Data Availability

The original contributions presented in the study are included in the article/Supplementary Material, further inquiries can be directed to the corresponding authors.
