# Supplementary material for: Off-label use of Baricitinib improves moderate and severe atopic dermatitis in China through inhibiting MAPK and PI3K/Akt/mTOR pathway via targeting JAK-STAT signaling of CD4+ cells
Source: Front Pharmacol. 2024 Feb 29;15:1324892. doi: 10.3389/fphar.2024.1324892 (PMC10937442; doi:10.3389/fphar.2024.1324892)
Supplement: Supplementary file 1 [file Table2.DOCX]

**Table of Contents**

**Supplementary methods and materials** 2

1. Histology and Immunohistochemical Staining 2
2. Western Blot 2
3. Assessment of cytokines and chemokines 3

**Supplementary figures** 4

Figure S1 4

Figure S2 5

Figure S3 6

Figure S4 6

Figure S5 7

**Supplementary methods and materials**

**1. Histology and Immunohistochemical Staining**

Tissue at skin lesions in moderate and severe AD patients and mice ears were fixed with 10% formaldehyde for 24h, embedded in paraffin, and cut into 4 µm-thick slices. The sections were stained with hematoxylin and eosin staining for routine histopathological examination and were evaluated using a light microscope (Leica, Germany) at 100**×** magnification. The epidermal thickness was measured by ImageJ (https:// imagej.nih.gov/ij/). Sections of skin lesions in AD patients were autoclaved in 0.5 mol/l sodium citrate for 5 min to repair the antigen and then were incubated in 1% H_2_O_2_ for 20 min at room temperature. After incubation in blocking solution, the slides were incubated overnight at 4°C with mouse anti-CD3 antibody (1:1000; Proteintech), rabbit anti-c-JUN antibody (1:200, Bioworld Technology, Inc.), and rabbit anti-IL4 antibody (1:200, Affinity Biosciences). The slides were then incubated with secondary antibody goat anti-mouse &rabbit IgG (1:200; Abcam) for 60 min at room temperature. Nuclei were counterstained with 4,6-diamidino-2-phenylindole (DAPI; 1μg/ml, Sigma). The slides were then analyzed using a Leica Fluorescence Microscope microscope (Leica, Germany).

**2. Western Blot**

Protein expression levels were assessed using Western blot analysis. Epithelial primary cells and mouse ears were homogenized in RIPA lysis buffer (Beyotime, Shanghai, China) containing protease inhibitors and were then centrifuged at 4°C, 14000 × g for 3 min. Total soluble protein content was assessed using a Beyotime protein quantification kit (Beyotime, Shanghai, China). The total cell lysates were separated on SDS-PAGE gels and transferred to PVDF membranes (Millipore). The PVDF membranes were blocked with 5% bovine serum albumin. We performed immunoblotting analysis with primary antibodies at 1:500 dilution for 24h at 4°C: anti-phospho c-JUN, anti-phospho P70S6K (ribosomal S6 protein kinase), anti-phospho AKT), anti-phospho STAT3 (Signaling Sensor and Activator of Transcription 3) and anti-phospho JNK were from Bioworld Technology, Inc. Anti-c-JUN, anti JNK, anti-P70S6K, anti-AKT, anti-STAT3, and anti-GAPDH rabbit polyclonal antibodies were from Affinity Biosciences. This was followed by incubation with the HRP-conjugated secondary antibody (supplied by ZSGB-Bio) at a 1:5000 dilution for 2h. Protein bands were visualized by enhanced chemiluminescence using gel analysis software ChemiDoc™ Touch.

**3. Assessment of cytokines and chemokines**

The co-culture supernatant samples were analyzed using the MILLIPLEX Cytokine/Chemokine panel (Millipore Corporation). The kit can detect the following 45 cytokines/chemokines according to the manufacturer's instructions. The samples were analyzed using a Luminex laser-based fluorescent analytical test with the Luminex 200 system. The concentrations of cytokines and chemokines were determined from standard curves prepared on each plate and expressed as picograms per milliliter (pg/ml).

**Supplementary figures**


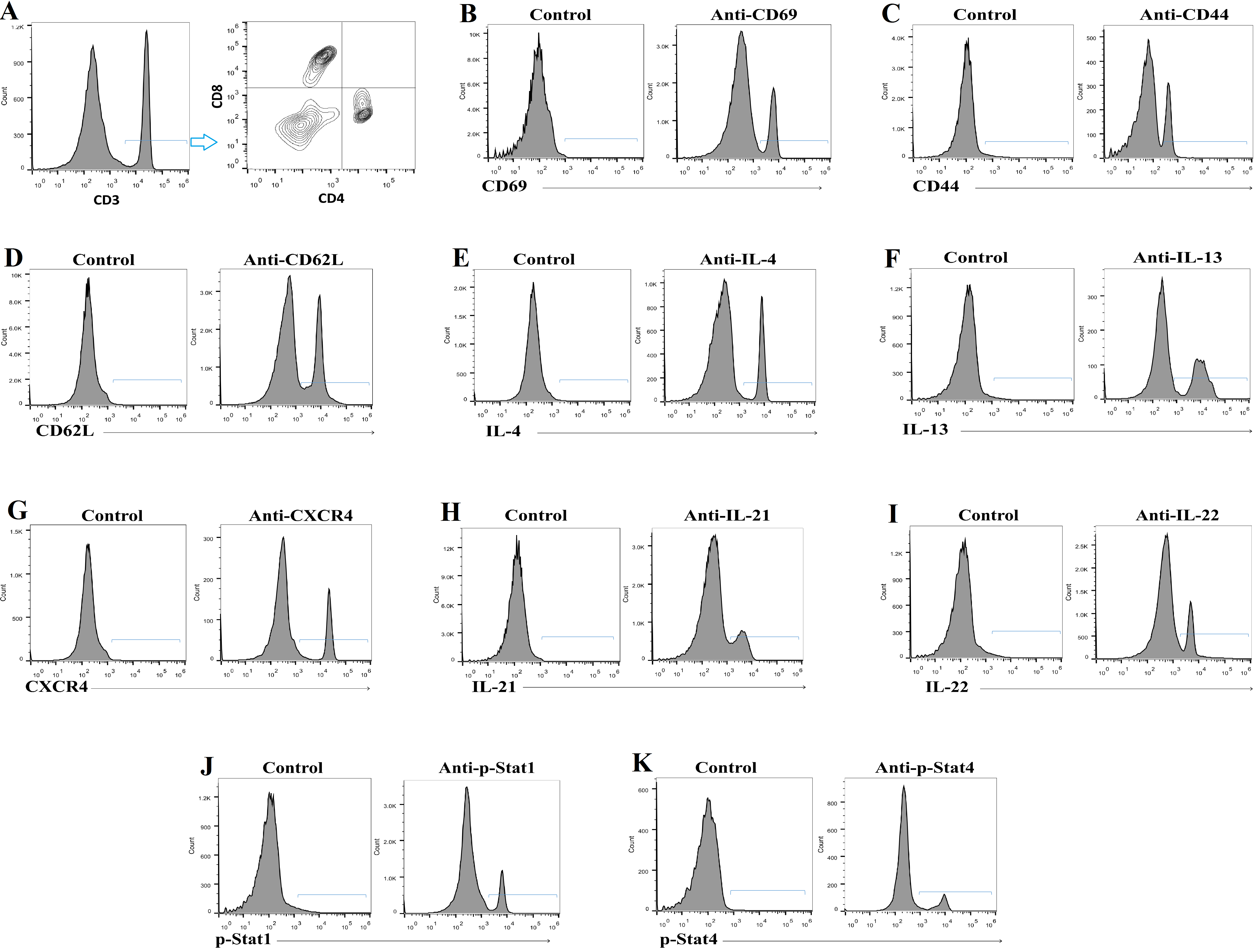


**Figure S1.** CD3^+^CD4^+^ and CD3^+^CD8^+^ T cells were gated (**A**), and the expression of CD69 (**B**),

CD44(**C**), CD62L(**D**), IL-4(**E**), IL-13(**F**), CXCR4(**G**), IL-21(**H**), IL-22(**I**), p-Stat1(**J**), p-Stat4(**K**) is displayed as open histograms with the control staining displayed as shaded histograms.

**Figure S2: Patient disposition:**71 patients with initial moderate and severe atopic dermatitis attending the Department of Dermatology at the First Hospital of Chongqing Medical University were screened, of whom 46 were enrolled. The most common reason (25%) for screen failure was not meeting the eligibility criteria. 10 patients (21.7%) discontinued treatment, and the most common reason for study discontinuation was patient withdrawal (5 patients [50%]). Ultimately 23 patients wholeheartedly completed the 4-week treatment period.


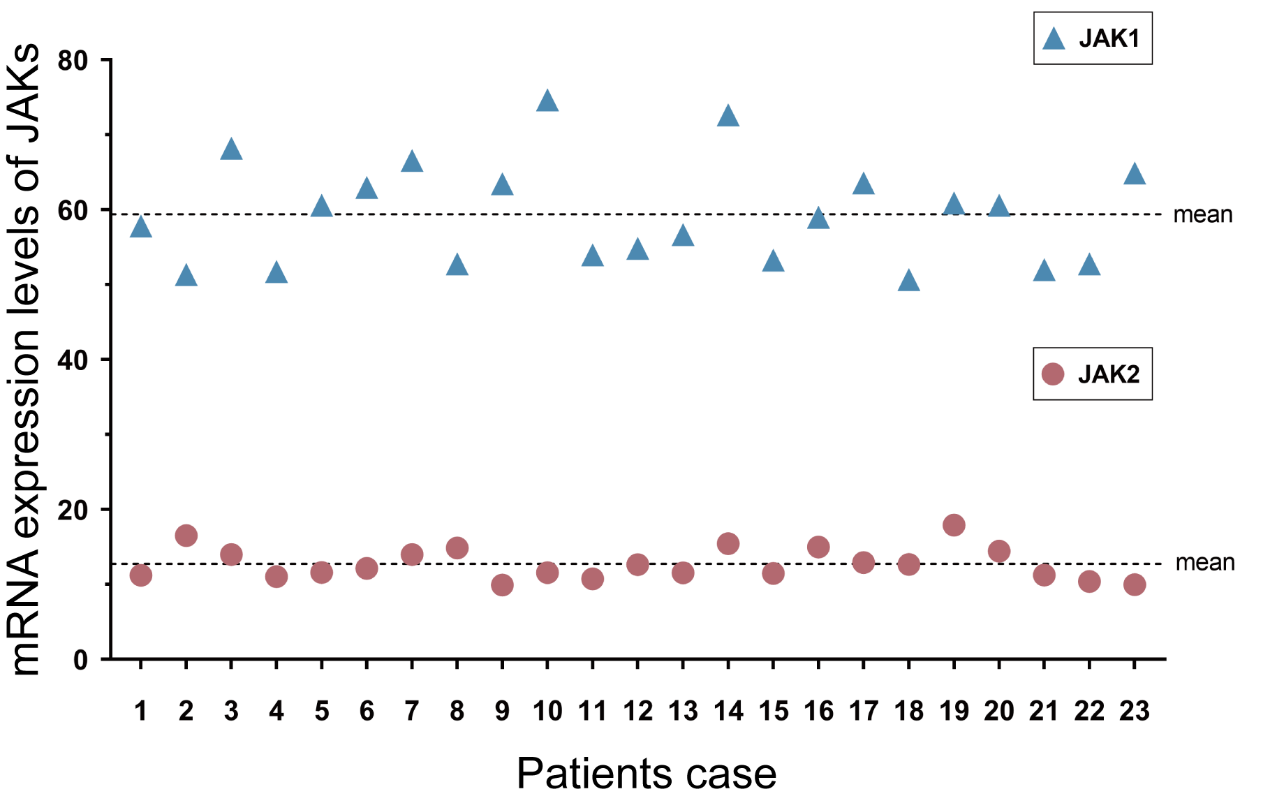


**Figure S3.** mRNA expression of JAK in AD patients(n=23). The mean expression value of JAK1/2 was set as a threshold value.23 AD patients were divided into a low JAK1/2 group (n=11/15) and a high JAK1/2 group (n=12/8) according to the threshold. Threshold of JAK1 = 59.37, threshold of JAK2 = 12.74.

**Figure S4:** Comparison of transcriptional profiles of inhibitory receptors genes, stimulatory molecules genes, transcription factors genes, cytokines genes, effector molecules genes and chemokines genes in peripheral blood CD8^+^ T cell subsets from AD patients (n=23) before and after baricitinib treatment. Heat map showing differentially expressed genes with fold change differences.


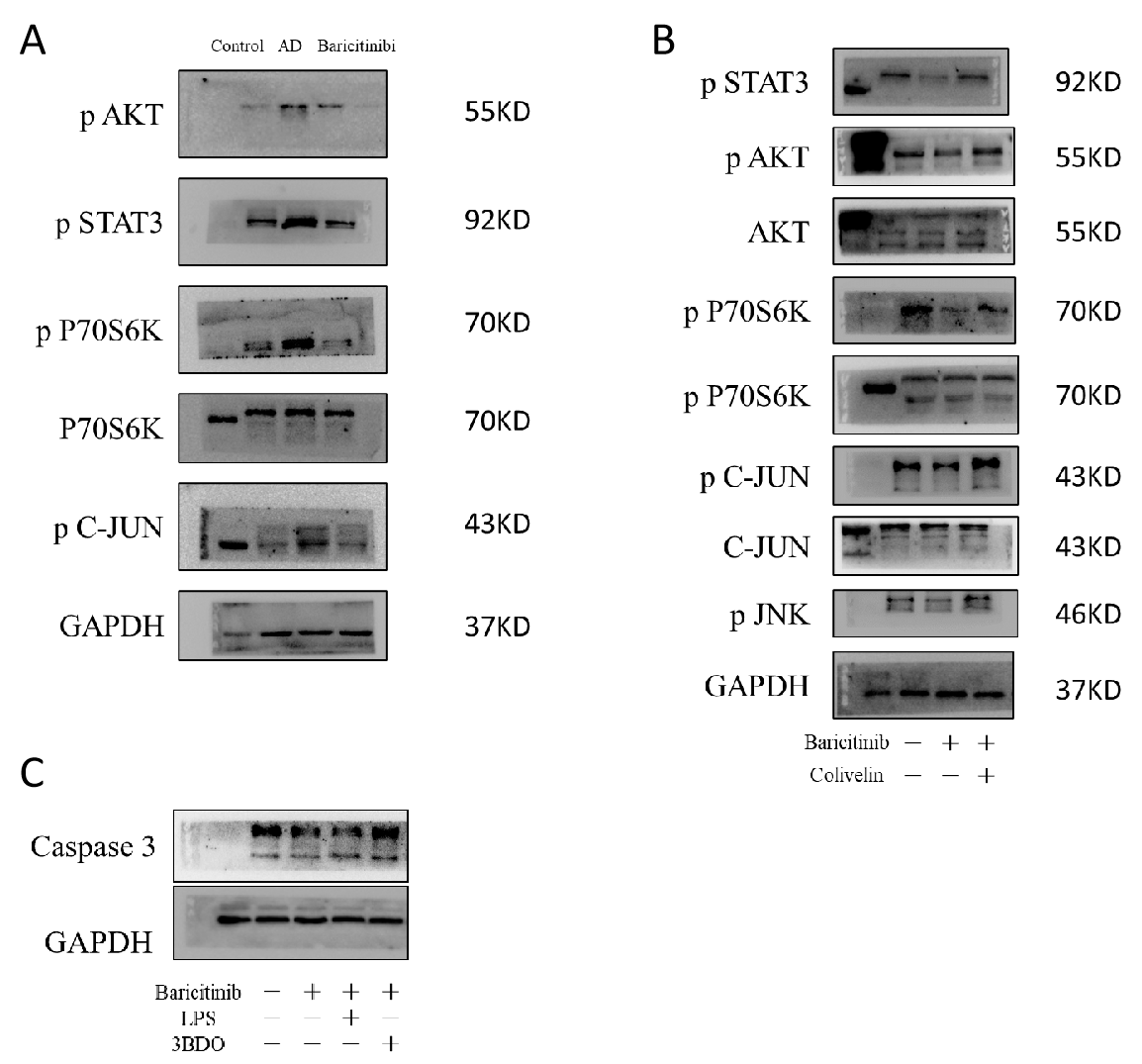


**Figure S5:** The full blots of Western blot in Figure6(**C**) and Figure7(**A, D**).
